# Deep-Sea Anemones Are Prospective Source of New Antimicrobial and Cytotoxic Compounds

**DOI:** 10.3390/md19120654

**Published:** 2021-11-24

**Authors:** Aleksandra Kvetkina, Elena Kostina, Irina Gladkikh, Victoria Chausova, Ekaterina Yurchenko, Irina Bakunina, Mikhail Pivkin, Stanislav Anastyuk, Roman Popov, Margarita Monastyrnaya, Emma Kozlovskaya, Marina Isaeva, Pavel Dmitrenok, Elena Leychenko

**Affiliations:** 1 G.B. Elyakov Pacific Institute of Bioorganic Chemistry, Far Eastern Branch, Russian Academy of Sciences, 690022 Vladivostok, Russia; irinagladkikh@gmail.com (I.G.); v.chausova@gmail.com (V.C.); dminae@mail.ru (E.Y.); bakun@list.ru (I.B.); oid27@mail.ru (M.P.); sanastyuk@piboc.dvo.ru (S.A.); rs.popov@outlook.com (R.P.); rita1950@mail.ru (M.M.); kozempa@mail.ru (E.K.); issaeva@gmail.com (M.I.); paveldmt@piboc.dvo.ru (P.D.); leychenko@gmail.com (E.L.); 2 A.V. Zhirmunsky National Scientific Center of Marine Biology, Far Eastern Branch, Russian Academy of Sciences, 690041 Vladivostok, Russia; cnidopus@mail.primorye.ru

**Keywords:** Actiniaria, Corallimorpharia, tentacle extracts, hemolytic activity, cytotoxicity, antimicrobial activity, antifungal activity, enzyme-inhibitory activity, sea anemone toxins

## Abstract

The peculiarities of the survival and adaptation of deep-sea organisms raise interest in the study of their metabolites as promising drugs. In this work, the hemolytic, cytotoxic, antimicrobial, and enzyme-inhibitory activities of tentacle extracts from five species of sea anemones (Cnidaria, orders Actiniaria and Corallimorpharia) collected near the Kuril and Commander Islands of the Far East of Russia were evaluated for the first time. The extracts of *Liponema brevicorne* and *Actinostola callosa* demonstrated maximal hemolytic activity, while high cytotoxic activity against murine splenocytes and Ehrlich carcinoma cells was found in the extract of *Actinostola faeculenta*. The extracts of *Corallimorphus* cf. *pilatus* demonstrated the greatest activity against Ehrlich carcinoma cells but were not toxic to mouse spleen cells. Sea anemones *C.* cf. *pilatus* and *Stomphia coccinea* are promising sources of antimicrobial and antifungal compounds, being active against Gram-positive bacteria *Bacillus subtilis*, *Staphylococcus aureus*, and yeast *Candida albicans*. Moreover, all sea anemones contain α-galactosidase inhibitors. Peptide mass fingerprinting of *L. brevicorne* and *C.* cf. *pilatus* extracts provided a wide range of peptides, predominantly with molecular masses of 4000–5900 Da, which may belong to a known or new structural class of toxins. The obtained data allow concluding that deep-sea anemones are a promising source of compounds for drug discovery.

## 1. Introduction

Deep-sea organisms, including sea anemones, are a rich source of biologically active compounds. They survive under extreme conditions in the absence of light, low levels of oxygen, and intensely high pressure that necessitate a diverse array of biochemical and physiological adaptations that may affect their gene regulation, primary metabolic pathways, and, consequently, their secondary metabolites [1,2]. Sea anemones of orders Actiniaria and Corallimorpharia are widespread in the world’s oceans, particularly in the Pacific Ocean, and they live from the intertidal zone to depths of over 10 km, including the maximal depths of the Mariana and Philippine Trenches [3].

Despite the great interest toward the sea anemones as producers of biologically active compounds, the venom composition of deep-sea anemones remains poorly understood. According to the ToxProt database [4], 252 peptide and protein toxins from 47 species of sea anemones are currently known, which covers less than 4% of all known species (≈1100). This makes it possible to consider unexplored and little-studied species to be sources of new biologically active protein compounds, many of which undoubtedly have pharmacological potential. Sea anemone venoms are a complex mixture of peptide and protein components, such as actinoporins [5,6,7,8,9], phospholipases A2 [10,11], neurotoxins [12], and enzymes and their inhibitors [13,14].

Actinoporins are one of the major components of sea anemone venoms. They are membranolytic polypeptides (20 kDa) that allow for them to exhibit anticancer, antibacterial, cardio-stimulating, and histaminolytic activities [15,16,17,18]. Some were used to create immunotoxins for anticancer therapy [19,20,21,22,23,24]. Neurotoxins (3–6 kDa) are one of the largest and best-characterized groups of sea anemone venom peptides. They interact with a wide range of ion channels, including voltage-gated sodium (Nav), potassium (Kv) channels, acid-sensing ion channels (ASICs), and transient receptor potential (TRP) channels. Neurotoxins acting on Nav (5 kDa) and Kv (4–6 kDa) channels, along actinoporins, predominate in the sea anemone venom and are the most studied groups of peptides [12,25,26]. Some Kunitz peptides (6 kDa), besides protease inhibition, are also able to block the activity of Kv channels [27,28,29] and modulate or block currents through TRPV1 channel [30,31,32,33], demonstrating anti-inflammatory [34,35,36], analgesic [37,38,39], and neuroprotective activity [40,41,42]. Recently, APETx-like peptides (4.5 kDa) modulating the action of ASICs [43,44,45,46] and defensin-like peptides modulating the transient receptor potential ankyrin 1 (TRPA1) channel (4.7 kDa) [47,48] have been discovered. Sea anemone toxins are mostly used as molecular tools to study the structural features and functional activity of ion channels, and they are pharmacological agents for the treatment of various diseases [49].

Traditionally, the isolation of bioactive peptides was conducted from sea anemone body tissue, tentacles, and mucus by the extraction and chromatographic separation approaches. In the last few decades, omics technologies such as genomics, transcriptomics, proteomics, and metabolomics have been in use for the venom component studies to better understand the ecology and defense strategies and discover biotechnological properties. Proteomic analysis coupled with mass spectrometry allows for estimating both the quantitative and qualitative peptide composition, and the tissue distributions of numerous peptides, and thus to assume their possible function in the sea anemone [50,51]. Such an approach was applied to study of venom components of *Bunodosoma cangicum* [52], *Bunodosoma granuliferum* [53], *Anemonia viridis* [54], *Heteractis magnifica* [55], *Homostichanthus duerdeni* (=*Stichodactyla duerdeni*) [56], and others [57].

In this work, we screened aqueous and ethanol extracts from the tentacles of deep-sea anemones collected from the Sea of Okhotsk, the Bering Sea, and the adjacent waters of the Pacific Ocean for the biologically active peptides with hemolytic, cytotoxic, antimicrobial, and enzyme-inhibitory activities. Using size-exclusion chromatography and mass spectrometry, we propose the peptide composition of previously unexplored *Liponema brevicorne* and *Corallimorphus* cf. *pilatus*.

## 2. Results

### 2.1. Identification of Deep-Sea Anemone Species

**Figure 1:**
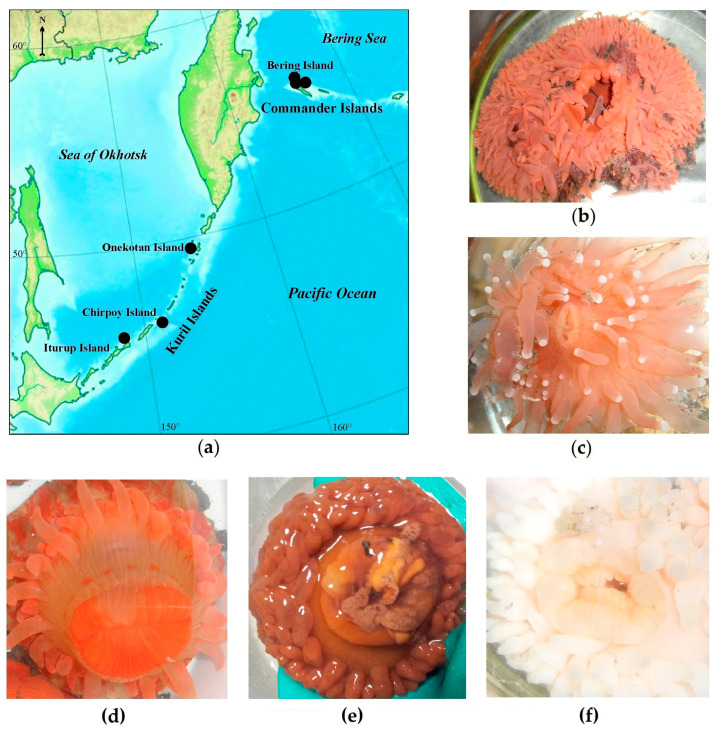
(**a**) Sampling area of sea anemones (**b**) *L. brevicorne*, (**c**) *C.* cf. *pilatus*, (**d**) *S. coccinea*, (**e**) *A. faeculenta*, and (**f**) *A. callosa* along the insular shelf and slope of the Kuril and Commander Islands of the Russian Far East.

Specimens of five deep-sea anemone species were collected from the Bering Sea and the Sea of Okhotsk in depths of 146–455 m (Table 1; Figure 1). The sea anemones were identified on the basis of the morphological description and phylogenetic markers. According to morphological features, four sea anemones were identified as *Actinostola callosa* (Verrill, 1882) (Figure 1f), *Actinostola faeculenta* (McMurrich, 1893) (Figure 1e), *Stomphia coccinea* (Müller, 1776) (Figure 1d) from the Actinostolidae family, and *L. brevicorne* (McMurrich, 1893) (Figure 1b) from Liponematidae family belonging to order Actiniaria, and one as *C.* cf. *pilatus* (Fautin, White et Pearson, 2002) (Figure 1c) belonging to order Corallimorpharia. Morphological features of *C.* cf. *pilatus* include its pinkish, short body with shallow longitudinal furrows; its oral disk has salient lips around the slit mouth and long not retractile tentacles with white acrospheres. *L. brevicorne* has a reddish–orange–pinkish, short, smooth body, hidden by an oral disc, the margin of which extends up to the level of a pedal disc and even the substrate; at living specimens, the animal resembles a low, tentacle-covered dome. Special features of *A. callosa* among the Actinostolidae family are the tentacle amount (up to 380), the different lengths of the inner and outer tentacles, and the presence of juvenile polyps (“giant larvae”) in the gastral cavity. *A. faeculenta* can be relatively light with a purple–brownish shade, the upper part of the column and very short tentacles (up to 200) are much darker than the rest of the body, while the cup-shaped column is very thick with tubercles. *S. coccinea* is reddish–cream–orange with orange stripes and has a cylindrical, smooth body with short tentacles (up to 82).

Sea anemones species were genetically identified by a comparison of sequences of such genetic markers as the nuclear 18S ribosomal RNA (18S) gene and internal transcribed spacer (ITS) gene region, and the mitochondrial cytochrome c oxidase subunit 1 (COI) gene with those available in the GenBank database. Approximately 1500 bp fragments of the 18S gene, 500–900 bp of the ITS gene region, and 600 bp fragments of the COI gene were successfully amplified from all sea anemone specimens except *C.* cf. *pilatus*; therefore, the identification of the sea anemone species was only based on morphological description. BLAST searches of all sequences revealed high identity values (98–100%) with sea anemone species in GenBank belonging to the same families as studied ones.

**Figure A1:**
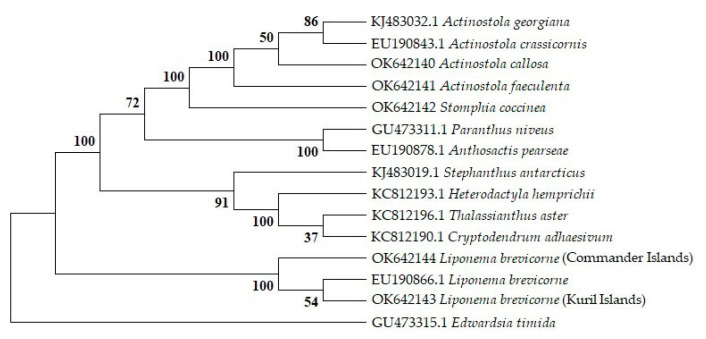
Neighbor-joining phylogenetic tree of sea anemones from suborder Nynantheae inferred from 18S gene sequences. NJ tree was constructed by MEGA X. The evolutionary distances were computed using the p-distance method. Bootstrap values (%) of 1000 replications are shown at internal nodes.

The phylogenetic analysis of the 18S (Appendix A Figure A1), COI, and ITS (Figure S1) gene sequences confirmed that the sea anemones *A. callosa*, *A. faeculenta*, and *S. coccinea* fall into the group of the Actinostolidae family to which they belong, while *L. brevicorne* specimens form a distinct group of the Liponematidae family. Thus, four sea anemones were identified on the basis of both morphological and genetic studies, while *C.* cf. *pilatus* was confirmed by only morphology.

### 2.2. Sea Anemone Tentacle Extraction

From the homogenized tentacles of every collected sea anemone sample, aqueous and ethanol extracts were prepared and incubated for 24 h at 4 °C. The weights of total proteinaceous compounds were from 2 to 15 mg (Table 1).

### 2.3. Determination of Biological Activity of Sea Anemone Extracts

Aqueous and ethanol extracts of the sea anemones were tested for hemolytic, cytotoxic, antibacterial, antifungal, and enzyme-inhibiting activities. Sea anemones *A. faeculenta*, *L. brevicorne*, and *C.* cf. *pilatus* were studied for the first time to identify biologically active compounds.

#### 2.3.1. Hemolytic Activity

All the aqueous extracts of the animals except *L. brevicorne* and *C.* cf. *pilatus*, collected near the Commander Islands, as well as ethanol extracts of *L. brevicorne* (Commander Islands) and *C.* cf. *pilatus* (Kuril Islands) demonstrated hemolytic activity. The aqueous extracts of *L. brevicorne* collected on the shelf of the Kuril Islands (8.8 µg/mL) and *A. callosa* (7.6 µg/mL), and ethanol extract of *L. brevicorne* collected near Commander Islands (7.6 µg/mL) showed maximal hemolytic activity (Table 2), while the aqueous extract of *A. faeculenta* and ethanol extract of *C.* cf. *pilatus* collected near Kuril Islands possessed weak activity against murine erythrocytes.

#### 2.3.2. Cytotoxic Activity

Both aqueous and ethanol extracts were tested for cytotoxic activity against murine splenocytes and Ehrlich carcinoma cells. The most extracts exhibited toxic activity against Ehrlich carcinoma cells and did not influence spleen cells viability (Table 2). The exceptions were the aqueous extract of *L. brevicorne* collected near Kuril Islands and ethanol extract of *A. faeculenta*, which possessed cytotoxic activity against murine splenocytes at concentration of 8.8 μg/mL and 9.3 μg/mL, respectively. The ethanol extract of *A. faeculenta* at the same concentration also exhibited cytotoxic activity against Ehrlich carcinoma cells (30%), while the aqueous extract at concentration of 35.5 μg/mL resulted in the death of 50% of Ehrlich carcinoma cells. The ethanol extracts of *C.* cf. *pilatus* collected near both the Kuril and Commander Islands also showed cytotoxic activity against Ehrlich carcinoma cells, with the extract of *C.* cf. *pilatus* from a slope of the Chirpoy Island (Kuril Islands) showed the greatest activity. Its components at a concentration of 9 μg/mL caused the death of 70% of tumor cells (Table 2).

#### 2.3.3. Antimicrobial Activity

The aqueous extracts of Kuril species *S. coccinea* and *C.* cf. *pilatus* at concentrations of 8.5 and 10 μg/mL, respectively, demonstrated a non-specific antimicrobial activity both against the Gram-positive bacteria *B. subtilis* and *S. aureus*, and pathogenic yeast *C. albicans*. The aqueous extract of sea anemone *L. brevicorne* also collected near the Kuril Islands only inhibited the growth of *B. subtilis* colonies at a concentration of 4.6 μg/mL (Table 2). Both *A. faeculenta* and *A. callosa*, as well as *L. brevicorne* and *C.* cf. *pilatus* collected near Commander Islands were not active against the Gram-positive bacteria. Among ethanol extracts, only *A. callosa* possessed antimicrobial activity; at a concentration of 12.5 μg/mL, it inhibited the growth of *S. aureus*. No extracts showed antibacterial activity against Gram-negative bacteria *Escherichia coli* and *Pseudomonas aeruginosa*.

#### 2.3.4. Enzyme-Inhibiting Activity

All extracts were tested for the presence of the inhibitors of trypsin, α-galactosidase, and its mutant with a substitution of C494N. The researches of serine protease inhibitors, particularly trypsin, from sea anemones have been conducting during the last 40 years and are of great interest for biomedicine [27,34,35,41,58,59]. α-Galactosidases (α-D-galactoside galactohydrolases, EC 3.2.1.22) have been known to catalyze the hydrolysis of non-reducing terminal α-D-galactose (Gal) from α-D-galactosides, galactooligosaccharides, and polysaccharides that serve as a source of carbon and energy for the organism’s growth. These enzymes are widespread among terrestrial plants, animals, and microorganisms, particularly in marine bacteria. Earlier the low molecular weight inhibitors of α-galactosidases were found in marine sponges *Monanchora pulchra* [60] and three species of *Dysidea* genus [61]. On the bases of these data, it has been proposed that sea anemones also have α-galactosidase inhibitors. The substitution of the key residue Cys494 to Asn in the active site of the enzyme results in decreasing the efficiency of retaining the affinity of the enzyme to standard substrate that allows identifying the competitive inhibitors and excluding allosteric inhibition [62]. Neither the aqueous nor the ethanol extracts of animals showed inhibitory activity against serine protease trypsin. All ethanol extracts inhibited the activity of α-galactosidase and its mutant C494N to a varying degree, except the extract of *L. brevicorne* from the shelf of the Commander Islands, which enhanced α-galactosidase activity (Table 3). The maximal inhibitory effect (up to 94% in relation to the enzyme) was observed for the ethanol extract of *L. brevicorne* (4.6 μg/mL) from the shelf of the Kuril Islands. The ethanol extract of *A. faeculenta* was less effective; at a concentration of 8.2 μg/mL, it inhibited α-galactosidase (80%) and C494N mutant (70%) activity. The other extracts inhibited enzyme activity in the range of 39–63%. All aqueous extracts increased α-galactosidase activity in the range of 115–174% (Table 3), which is interesting due to there being no sufficient information about α-galactosidase activators.

### 2.4. Identification of Peptide Compositions in L. brevicorne and C. cf. pilatus Extracts

**Figure 2:**
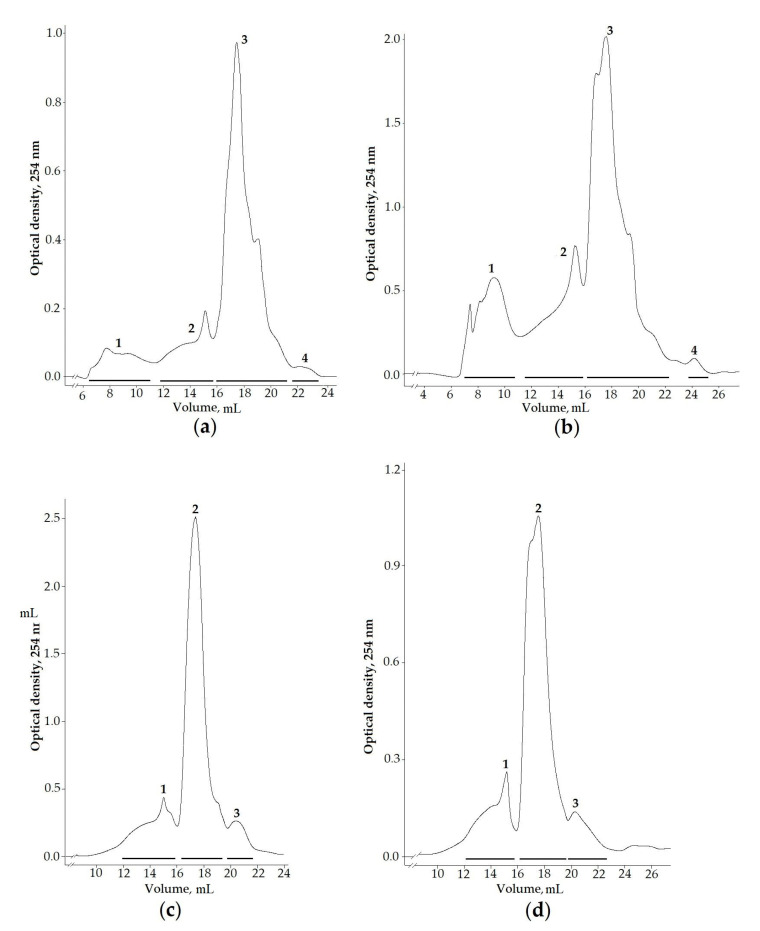
Gel filtration profiles of (**a**,**b**) aqueous and (**c**,**d**) ethanol extracts of *L. brevicorne* and *C.* cf. *pilatus*, respectively. Samples were dissolved in 0.1 M ammonium acetate buffer solution, pH 6.0, and applied onto a Superdex Peptide 10/30 column equilibrated with the same buffer solution. The flow rate was 0.2 mL/min, and the fraction volume was 1.0 mL.

To identify the peptide components of sea anemone venoms, the aqueous and ethanol extracts of *L. brevicorne* and *C.* cf. *pilatus* collected near the Kuril Islands were selected for chromatographic division due to them exhibiting enzyme-inhibiting, antimicrobial, and cytotoxic activities. Moreover, the venom composition of both deep-sea anemone species has never been studied before. The separation of targeted extracts on a size-exclusion column allowed obtaining four fractions from both *L. brevicorne* and *C.* cf. *pilatus* aqueous extracts (Figure 2a,b) and three fractions from *L. brevicorne* (Figure 2c) and *C.* cf. *pilatus* (Figure 2d) ethanol extracts. Both the aqueous and the ethanol extracts of the sea anemones had similar elution profiles.

All the obtained fractions were processed using MALDI-TOF/TOF. A total of 70 peptides with molecular masses of 1458–10,395 Da, and 83 peptides with molecular masses of 1298–9812 Da were identified in the aqueous extracts of *L. brevicorne* and *C.* cf. *pilatus*, respectively (Table 4). The ethanol fractions of *L. brevicorne* contained 50 peptides with molecular masses from 1527 to 10,846 Da, while *C.* cf. *pilatus* fractions contained 43 peptide masses from 2984 to 9808 Da.

**Figure 3:**
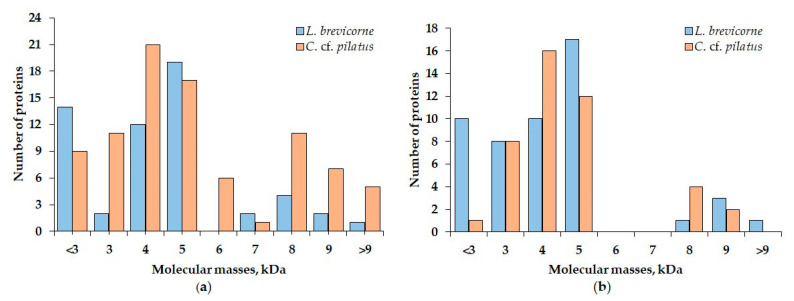
Distribution of components identified in (**a**) aqueous and (**b**) ethanol extracts of *L. brevicorne* and *C.* cf. *pilatus*.

The comparison of obtained MALDI spectra revealed the presence of peptides with closely related molecular masses between *L. brevicorne* and *C.* cf. *pilatus*. Such examples were peptides with molecular masses 3783 and 3781, 5188 and 5184, 5554 and 5552, and 5884 and 5888 Da from aqueous extracts, and 2982 and 2984, 3052 and 3053, 5381 and 5378, 5550 and 5546, and 5555 Da from ethanol extracts of *L. brevicorne* and *C.* cf. *pilatus*, respectively (Table 4). Two peptides had identical molecular mass (5774 and 5275 Da) in the extracts of both sea anemones. According to Figure 3, the peptide molecular mass distribution in aqueous extracts of *L. brevicorne* and *C.* cf. *pilatus* is more diverse than that of ethanol extracts. The most prevalent *L. brevicorne* peptide masses were in the range of 5000–5900 Da, while *C.* cf. *pilatus* contained peptides with the highest frequency between 4000 and 5900 Da.

The deeper identification of extract components was carried out with fraction 3 of *L. brevicorne* ethanol extract using tandem mass spectrometry (MS/MS). Spectra from a tandem MS experiment of the alkylated fraction were searched against the Actiniaria protein database from UniprotKB (50,697 sequences, downloaded on 24 September 2021 from www.uniprot.org (accessed on 18 October 2021)). From seven validated protein fragments, only three structural-like proteins, two enzymes, and two uncharacterized proteins were identified (Table S1), while toxin-like peptides were not found. The tryptic digestion of the peptide fraction did not produce additional results, which could be related to the toxins being minor components of *L. brevicorne* venom or the absence of some information about *L. brevicorne* venom components, which is of great both fundamental and applied interest.

## 3. Discussion

Due to the extreme environment, deep-sea anemones need to adapt their primary metabolic pathways and venom compositions [1,2]. *S. coccinea*, *A. callosa*, *A. faeculenta*, and species of the *Corallimorphus* genus are subtidal–bathyal species (*A. callosa* lives up to 2 km depth). Sea anemone *S. coccinea* also prefers bouldery–gravelly substrata, shell debris, and sometimes, it occurs in silted seabed, but it can detach from the substratum and swim. Species of *Actinostola* can be found in sandy or silted bottoms and hard surfaces, but it is not always attached to the substratum like *S. coccinea*. Sea anemone *C.* cf. *pilatus* attach themselves to the bedrock outcrops, boulders, and other hard surfaces, often with sandy or silted overlay. Sea anemone *L. brevicorne* inhabits both the bathyal and upper abyssal zones (above 4 km depth), and this species is sometimes found on the shelf [63,64]. This sea anemone lives unattached on sandy or muddy bottoms, sometimes with an admixture of gravel, small boulders, or fragments of shell debris.

The taxonomic identification of sea anemones is carried out by morphological description, which can be difficult due to some species being virtually identical in appearance and distinguished by only one or two morphological features. Moreover, the morphological analysis of sea anemones is time consuming and requires considerable expertise [65]. An approach based on appropriate molecular genetic methods is widely used. All collected sea anemone specimens were identified by both approaches. According to the morphological features and comparison of nuclear and mitochondrial markers, *A. callosa*, *A. faeculenta*, *S. coccinea*, and *L. brevicorne* confirmed the species affiliation (Figure A1), while *C.* cf. *pilatus* was only morphologically identified.

Similarly to previous research of extracts of sea anemones inhabiting the southern part of the Sea of Okhotsk, near Sakhalin Island [13], the aqueous extracts of animals studied in this work and ethanol extracts of Commander *L. brevicorne* and Kuril *C.* cf. *pilatus* demonstrated hemolytic activity (Table 2). The hemolytic activity of aqueous extracts is probably associated with the presence of pore-forming toxins, in particular actinoporins. Moreover, sea anemones near the Kuril and Commander Islands maintained a trend of decreasing the hemolytic activity of aqueous extracts as compared to that of tropical species. This is probably associated with the different habitat conditions of the animals: the temperature of sea water, the amount of food, and the presence of possible predators [7]. Therefore, actinoporins produced by sea anemones *Oulactis orientalis* and *Metridium senile* collected in the Sea of Japan [13,66] demonstrated hemolytic activity by a factor of 100 lower than that of tropical species, such as *Heteractis crispa* (= *Radianthus macrodactylus*) [6,16].

The cytotoxic activity of aqueous and ethanol extracts of sea anemones against murine splenocytes and Ehrlich carcinoma cells probably indicates the presence of neurotoxins or pore-forming toxins in the studied species. Actinoporins RTX-A [67] and Hct-S3 [18] from *H. crispa* showed anticancer activity, and StI from *Stichodactyla helianthus* [19], EqtII, and its mutant I18C from *Actinia equina* [20,21], gigantoxin-4 from *Stichodactyla gigantea* [23], and FraC from *Actinia fragacea* [24] were used to create immunotoxins that destroy tumor cells. The ethanol extracts of *C.* cf. *pilatus* collected both near the Kuril and the Commander Islands demonstrated cytotoxic activity against Ehrlich carcinoma cells but were not toxic to mouse spleen cells, which indicates that these animals are promising sources of compounds that could influence negatively cancer cells.

The presence of the antimicrobial activity of *L. brevicorne*, *S. coccinea*, and *C.* cf. *pilatus* extracts against Gram-positive bacteria *B. subtilis* and *S. aureus* as well as pathogenic yeast *C. albicans* is interesting (Table 2). The presence of numerous defensive components, such as antimicrobial peptides for protection against microorganisms including pathogens, whose action they are constantly exposed to in habitats due to the lack of an adaptive immune system in sea anemones, is no coincidence [68,69]. Antimicrobial peptides were isolated from sea anemones *Urticina eques*, Ueq 12-1 [48], *Anemonia sulcata*, ATX II [70], and *Urticina crassicornis*, crassicorin-I and-II [71]. Thus, sea anemones *S. coccinea*, *C.* cf. *pilatus*, and *L. brevicorne* are promising cnidarians for the search and isolation of new antimicrobial compounds.

Trypsin inhibitors were present in the extracts of *S. coccinea* and *A. callosa* collected from the Sea of Okhotsk, near southern Sakhalin Island [13]. The absence of protease inhibitors in the extracts of sea anemones collected on the insular shelf and slope of the Kuril and Commander Islands probably indicates a scarce diversity of food sources and, consequently, the low activity of proteolytic enzymes. Nevertheless, ethanol extracts inhibited the activity of α-galactosidase and its mutant C494N to a varying degree, except for the extract of *L. brevicorne* from the shelf of the Commander Islands, which enhanced its activity (Table 3). Moreover, all aqueous extracts showed α-galactasidase-activating activity, which might indicate the presence of effectors of this enzyme in the sea anemones. The blocking of biochemical processes in which glycosidases are involved through powerful selective inhibitors underlies the treatment of a number of infectious diseases, malignant neoplasms, and genetic disorders [72], while effectors can be successfully applied in biotechnology and biomedicine to reduce the amount of enzymes used. Therefore, the search for new natural effectors or inhibitors of these enzymes, about which almost no data are available, is quite relevant.

The extracts of *L. brevicorne* and *C.* cf. *pilatus* collected near Kuril Islands were chosen for further analysis due to exhibiting enzyme-inhibiting, antimicrobial, and cytotoxic activities (Table 2 and Table 3). Moreover, to the best of our knowledge, the venoms of both sea anemone species have not been studied and are thereby interesting for the research of new biological active compounds. Through gel filtration chromatography of *L. brevicorne* and *C*. cf. *pilatus* extracts, we obtained a number of fractions, the peptide mass fingerprinting of which provided peptides in the range of molecular masses from 1298 to 10,846 Da (Figure 2; Table 4) but predominantly with molecular masses of 4000–5900 Da, which may belong to known or new structural toxins. At this stage of research, we cannot clearly conclude what compound is responsible for the manifestation of a particular type of activity. Moreover, MS/MS analysis of ethanol fraction 3 of *L. brevicorne* did not allow for identifying peptide toxins that are probably associated with toxins that are minor venom components or lacking any information about sea anemone venom compounds in databases. This creates the potential for further deeper investigation of the venom components of *L. brevicorne*. Further transcriptomic analysis will allow providing complete genetic information about the diversity of peptide toxins, obtaining recombinant analogs, and investigating their biological activities.

In conclusion, the comparison of the biological activity of the *L. brevicorne* and *C.* cf. *pilatus* extracts showed that sea anemones inhabiting the shelf and slope of the Kuril Islands have a wider spectrum of activities than that of animals collected near the Commander Islands. Such differences in the biological activity of compounds produced by one species of sea anemones could be explained by the difference in their habitat and environmental conditions: water temperature, depth, food diversity, the presence or absence predators, and other factors. Obtained data create a basis for the more in-depth study of peptides from the venom of sea anemones inhabiting the northwestern seas of the Pacific Ocean and allow for concluding that deep-sea anemones could be a promising source of compounds for biotechnology and biomedicine.

## 4. Materials and Methods

### 4.1. Sea Anemone Collection

The sea anemones *A. callosa* (Verrill, 1882), *A. faeculenta* (McMurrich, 1893), *S. coccinea* (Müller, 1776), *L. brevicorne* (McMurrich, 1893) from order Actiniaria, and *C.* cf. *pilatus* (Fautin, White et Pearson, 2002) from order Corallimorpharia were collected by a Sigsby trawl in the Sea of Okhotsk, the Bering Sea, and the adjacent waters of the Pacific Ocean to 500 m isobath, on the insular shelf and slope of the Kuril (Iturup, Chirpoy, and Onekotan islands) and Commander Islands (Bering Island) during cruises of the R/V Akademik Oparin no. 47 (2015) (Table 1; Figure 1).

### 4.2. DNA Extraction, Amplification, and Sequencing

To confirm the species identification of sea anemone samples, we used two nuclear markers, the 18S rRNA (18S) and ITS gene region, and one mitochondrial marker, cytochrome c oxidase subunit 1 (COI). Genomic DNA was extracted from sea anemone tissue using the MagJET Plant Genomic DNA Kit (Thermo Fisher Scientific, Waltham, MA, USA) according to the manufacturer’s protocol. PCR was conducted using GoTaq Flexi DNA Polymerase (Promega, Madison, WI, USA). The 18S gene was amplified using primer pair 18S_Hmag_Rev (5′-AAGGGCAGGGACGTAATC-3′) and 18S_Hmag_For (5′-GAAACTGCGAATGGCTCA-3′), which were designed on the basis of conservative 5′- and 3′-tereminal sequences of sea anemone 18S genes. The reaction profile was 95 °C for 180 s, 30 cycles of 94 °C for 20 s, 50 °C for 20 s, 72 °C for 120 s, and 72 °C for 600 s. The COI gene was amplified using primer pair COI_anemone_Rev (5′-CTGCYGGGTCAAARAAAG T-3′) and COI_anemone_For (5′-TGGAAKAGGRTCYGGTATGA-3′). The primers were designed on the basis of conservative 5′-and 3′-tereminal sequences of sea anemone COI genes belonging to families Actiniidae and Actinostolidae. The reaction profile was 95 °C for 180 s, 30 cycles of 94 °C for 20 s, 55 °C for 20 s, and 72 °C for 80 s, and 72 °C for 300 s. The ITS gene region was amplified using primer pair 18S-28S_Hmag_For (5′-GCCGAAAAGTTGTTCAAA-3′) and 18S-28S_Hmag_Rev (5′-TAAATTCAGCGGGTAGTC-3′), which were designed on the basis of conservative 5′-and 3′-tereminal sequences of the sea anemone ITS gene region. The reaction profile was 95 °C for 180 s, 30 cycles of 94 °C for 20 s, 50 °C for 20 s, 72 °C for 80 s, and 72 °C for 300 s. The amplified 18S and COI genes were purified with the Cleanup Mini Kit (Evrogen, Moscow, Russia) and sequenced on an Applied Biosystems SeqStudio Genetic Analyzer (Thermo Fisher Scientific, Waltham, MA, USA) using the Big Dye Terminator reagent kit, version 3.1. The amplified ITS genes were purified, cloned with the CloneJET PCR Cloning Kit (Thermo Fisher Scientific, Waltham, MA, USA), and transformed into Top 10 *Escherichia coli* cells (Invitrogen, Life Technologies) according to standard protocols. Plasmids containing the insertions were sequenced as described above. Gene sequences were deposited in GenBank under accession numbers OK642140-OK642144 for the 18S gene, OK638712-OK638716 for the COI gene, and OL329854-OL329860 for the ITS gene region.

### 4.3. Phylogenetic Analysis

The 18S, COI, and ITS gene sequences were aligned by MEGA X software version 11.0.9 [73] using Clustal W (for 18S, COI) and MUSCLE (for ITS) parameters. Sea anemone sequence homologs were searched in the GeneBank database using the BLASTN algorithm (http://www.ncbi.nlm.nih.gov/BLAST, accessed on 18 October 2021). Phylogenetic analysis was conducted using MEGA X software [73]. All sequence alignments were model-tested prior to tree constructions. A phylogenetic tree was constructed according to the neighbor-joining (NJ) algorithm using the p-distance model and pairwise deletion. The topologies of the trees were evaluated by 1000 bootstrap replicates.

### 4.4. Preparation of Aqueous and Ethanol Extracts

One-half of the tentacles of each animal were homogenized with cold water, and the other half were homogenized with cold 96% ethanol in a ratio of one part homogenate to three parts solvent. Extracts were filtered after 12 h and centrifuged at 10,000× *g* for 15 min at 4 °C. The supernatants of aqueous extracts were used for future analyses. Ethanol extracts were preliminarily distilled using the rotor evaporator (Buchi, Flawil, Switzerland) to remove ethanol, and the resulting supernatant was used for further separation.

### 4.5. Determination of Protein Concentration

The protein concentration was determined by the method of Lowry [74] in the extracts on a plate spectrophotometer BioMark xMark at λ = 750 nm. Bovine serum albumin was used as a standard.

### 4.6. Trypsin Inhibition Assay

The trypsin inhibitory activity of peptide fractions was tested through the standard procedure [75] using Nα-benzoyl-D,L-arginine p-nitroanilide (BAPNA) (Sigma Aldrich, St. Louis, MO, USA) as a substrate.

### 4.7. Assay of Enzymatic Activity of Recombinant α-Galactosidase and Its Mutant Form from Marine Bacterium Pseudoalteromonas KMM 701

Recombinant α-galactosidase and its C494N mutant form were obtained according to a previously described procedure [60,62]. Enzymes and substrate 4-nitrophenyl-α-D-galactopyranoside (Sigma-Aldrich, St. Louis, MO, USA) were dissolved in 0.05 M sodium phosphate buffer, pH 7.0. To estimate the influence of targeting extracts on enzymatic activity, 0.05 mL of an enzyme solution (0.02 ± 0.001 units of α-galactosidase or 0.002 unit of C494N mutant) was mixed with 0.025 mL of the extract (5 mg/mL) and incubated for 30 min at room temperature. Then, 0.075 mL substrate (stock concentration of 1.65·mM) was added. The reaction mixture was incubated at 20 °C for 4 min for α-galactosidase and 10 min for C494N. The reaction was stopped by the addition of 0.15 mL of 1 M Na_2_CO_3_. Hydrolytic activity of galactosidases was spectrophotometrically measured (ε_400_ = 18,300 M^−1^cm^−1^). One unit of activity (U) was determined as the amount of enzyme that released 1 µmol of *4*-nitrophenol per 1 min at 20 °C in 0.05 M sodium phosphate buffer at pH 7.0. After extract addition, residual enzymatic activity was measured as U/U_0_ and represented in percentage, where U_0_ is the standard activity of α-galactosidases without extracts.

### 4.8. Hemolytic Activity Assay

Hemolytic activity was detected in a 0.7% solution of mouse erythrocytes in a medium containing 0.9% NaCl, 1 mM KCl, and 10 mM glucose. Then, 0.01 mL of targeting extracts was mixed with 0.09 mL of erythrocyte suspension, and the mixture was incubated for 1 h at 37 °C. Hemoglobin level in the supernatant was spectrophotometrically measured at λ = 540 nm after the preliminary rapid cooling of the reaction mixture and its centrifugation to precipitate erythrocytes and their shadows. The optical density of the supernatant (0.8) of the control specimens, where the lysis of erythrocytes was induced by the addition of 0.01 mL 1% solution of holothurin A1 from sea cucumber *Eupentacta fraudatrix* [76], was assumed to be 100% hemolysis. Measurements were performed three times. Hemolytic activity was presented as minimal amount of protein (MC_100_) causing 100% hemolysis of red blood cells.

### 4.9. Assay of Cytotoxicity Effect against Murine Ascite Ehrlich Carcinoma Cells In Vitro

The museum tetraploid strain of ascite Ehrlich carcinoma cells was received from the N.N. Blokhin Russian Cancer Research Center. Ehrlich carcinoma cells were injected into the mouse abdominal cavity and harvested on the 7th day after inoculation. Mice were anaesthetized with diethyl ether, their abdominal cavity was rapidly opened, and ascitic fluid with cancer cells was collected with a syringe. Cells were washed three times with phosphate saline buffer (PBS), pH 7.4 using at least 10 volumes of a washing solution by centrifugation (1000 rpm) for 5 min and resuspended with RPMI-1640 medium (1−2 × 10^6^ cells/mL). Extracts (20 µL) were added to 180 µL of cell suspension and incubated in a 96-well plate at 37 °C for 24 h. Cell viability was measure by MTT assay (Sigma-Aldrich, St. Louis, MO, USA) according to the manufacturer’s protocol. Cytotoxic activity was presented as a minimal concentration of extract, resulting in 100% cell death.

### 4.10. Assay of Cytotoxic Effect against Murine Spleen Cells

Murine splenocytes were resuspended in RPMI-1640 medium at a concentration of 2 × 10^6^ cells/mL. The studied extract (20 µL) was added to 180 µL of cell suspension and incubated in 96-well plate at 37 °C for 24 h. Cell viability was measured by MTT assay (Sigma-Aldrich, St. Louis, MO, USA) according to producer protocol. Cytotoxic activity was presented as a minimal concentration of extract, resulting in 100% cell death.

### 4.11. Antimicrobial Assay

The antimicrobial activity of extracts was determined by diffusion in an agar layer with holes [77]. The agar was autoclaved for 15 min at 121 °C, cooled to about 55 °C, and poured into sterile Petri dishes. A hole with an 8 mm diameter in the agar layer was produced with a sterile cork borer. Test cultures of microorganisms were sown as a lawn (approximately 10^6^ cells/mL). Holes were filled with 50 μL of test extracts, and Petri dishes were incubated for 12–48 h at 28 °C. The zone diameter of the absence of test culture growth around the holes was measured in millimeters. Test cultures of *S. aureus*, *B. subtilis* (Gram-positive or monoderm bacteria), *E. coli*, *P. aeruginosa* (Gram-negative or diderm bacteria), and *C. albicans* (unicellular microscopic fungi–yeast) were used.

### 4.12. Size-Exclusion Chromatography

Aqueous and ethanol extracts of tentacles isolated from the *L. brevicorne* and *C.* cf. *pilatus* (Kuril Islands) were lyophilized. The yield of dry protein from aqueous and ethanol extracts was 33 and 11 mg for *L. brevicorne*, and 18 and 37 mg for *C.* cf. *pilatus*, respectively. Dry protein mixtures were dissolved in distilled water and applied to a Superdex Peptide 10/30 (1 × 30 cm) column (Amersham Pharmacia Biotech AB, Staffanstorp, Sweden), equilibrated with 0.1 M ammonium acetate buffer solution, pH 6.0. The sample volume was 100 μL. Peptides were eluted with the same buffer solution at a flow rate of 0.2 mL/min; the volume of fractions was 1.0 mL. Fractions of one peak were combined.

### 4.13. Mass Spectrometry Analysis

The molecular weight of peptides in the eluted fractions was determined on an Ultraflex III MALDI-TOF/TOF time-of-flight mass spectrometer (Bruker Daltonics, Bremen, Germany) with a laser ionization source (SmartBeam, 355 nm). Sinapic acid (3,5-dimethoxy-4-hydroxycinnamic acid) was used as the matrix. Time-of-flight mass spectra were recorded in linear and reflection modes.

### 4.14. Sample Preparation for Proteomic Analysis

The protein fraction was processed with 10 mM dithiothreitol to reduce cysteine residues, alkylated with 50 mM iodoacetamide for 30 min in the dark at 37 °C, and digested in 50 mM ammonium bicarbonate buffer with trypsin (MS Grade, Pierce) overnight at 37 °C. Alkylated and/or digested fractions were applied on an Acclaim PepMap RSLC C18 column (75 μm × 150 mm, Thermo Scientific) with a cartridge-based trap C18 column μ-Precolumn (300 μm × 5 mm, Thermo Scientific) using an UltiMate 3000 RSLCnano System (Dionex, Sunnyvale, CA, USA) equipped with a pump module, a column compartment (NCS-3500 RS), and an autosampler (WPS-3000TPL RS). The injection volume was 20 μL. Samples were loaded onto the μ-Precolumn equilibrated in 1% aqueous acetonitrile containing 0.1% FA for 4 min at 4 µL/min; after that, samples were eluted onto the analytical column. The mobile phases consisted of water containing 0.1% FA as solvent A and acetonitrile containing 0.1% FA as solvent B. The gradient profile began with 4% B at 300 nL/min flow rate, increased to 8% B to 10 min, and then to 50% B from 10 to 60 min, from 50 to 99% B from 60 to 61 min, isocratic at 99% of eluent B to 70 min, and lastly decreased to 4% B to 71 min for column equilibration for 15 min. Eluted peptides were analyzed by a Bruker Impact II Q-TOF mass spectrometer (Bruker Daltonics, Bremen, Germany) equipped with a Captive Spray ionization source (Bruker Daltonics, Bremen, Germany). MS spectrum range was from 100 to 3000 *m/z*. Capillary voltage was set to 1500 V, and the drying gas was heated to 150 °C at a flow rate of 3 L/min. Collision-induced dissociation (CID) product ion mass spectra were recorded in auto-MS/MS mode, the threshold for precursor ion isolation was set to 1000, and active exclusion after the two spectra was used. Collision energy was automatically set from 23 to 65 eV according to the molecular masses of precursor ions chosen for fragmentation with an isolation window width of 3 Da. Nitrogen was used as the collision gas. The mass spectrometer was calibrated using the ESI-L Low Concentration Tuning Mix (Agilent Technologies, Santa Clara, CA, USA) under conditions recommended by the manufacturer. Lock-mass calibration with hexakis (1H,1H,3H-tetrafluoropropoxy) phosphazine (922.0098 *m/z* in positive mode; 966.0007 *m/z* in negative mode; Agilent Technologies, Santa Clara, CA, USA) was performed; a calibrant was applied to the inner wall of the air filter of CaptiveSpray source. The instrument was operated using otofControl (ver. 4.1, Bruker Daltonics, Bremen, Germany).

### 4.15. Tandem Mass Spectrometry Analysis

Peak lists obtained from MS/MS spectra were identified using Data Analysis Software ver. 4.4 (Bruker Daltonics, Bremen, Germany), OMSSA version 2.1.9 [78], Andromeda version 1.5.3.4 [79], MS Amanda version 2.0.0.17442 [80], MS-GF+ version release 22 March 2021 [81], and Comet version 2021.01 rev. 0 [82]. The search was conducted using SearchGUI version 4.0.41 [83]. Protein identification was conducted against a concatenated target/decoy [84] protein database consisted in the combination of the UniprotKB sequences from all organisms from the Actiniaria taxon (50,967 sequences, released 24 September 2021). Decoy sequences were created by reversing the target sequences in SearchGUI. Identification settings were as follows: no cleavage specificity; 0.07 Da as MS1 and 40.0 ppm as MS2 tolerances; fixed modifications: carbamidomethylation of C (+57.021464 Da); variable modifications: acetylation of protein N-term (+42.010565 Da), oxidation of M (+15.994915 Da), fixed modifications during the refinement procedure: carbamidomethylation of C (+57.021464 Da). Peptides and proteins were inferred from spectral identification results using PeptideShaker version 2.0.33 [85]. Peptide spectrum matches (PSMs), peptides, and proteins were validated at a 1.0% false discovery rate (FDR), which was estimated using decoy hit distribution.

## Figures and Tables

**Table 1:** Sea anemone habitat and protein weight of aqueous and ethanol extracts.

Sea Anemone	Habitat	Protein, mg	
		Aqueous Extract	Ethanol Extract
Order Actiniaria Family Actinostolidae			
*Stomphia coccinea*	The Sea of Okhotsk, Iturup Island, 45°44.4′ N, 148°33.4′ E, 263 m, gravel	8.5	14.5
*Actinostola callosa*	The Bering Sea, Bering Island, 55°25.2′ N, 165°49.8′ E, 207 m, silt	7.6	2.6
*Actinostola faeculenta*	The Bering Sea, Bering Island, 55°25.2′ N, 165°49.8′ E, 207 m, silt	8.3	9.3
Family Liponematidae			
*Liponema brevicorne*	The Sea of Okhotsk, Onekotan Island, 49°24.1′ N, 154°16.1′ E, 146 m, sand, shells	4.6	4.0
*Liponema brevicorne*	The Bering Sea, Bering Island, 55°18.5′ N, 166°31.4′ E, 153 m, silt	12.3	5.1
Order Corallimorpharia Family Corallimorphidae			
*Corallimorphus* cf. *pilatus*	The Pacific Ocean, Chirpoy Island, 46°21.1′ N, 150°59.0′ E, 455 m, gravel	10.0	9.0
*Corallimorphus* cf. *pilatus*	The Bering Sea, Bering Island, 55°25.7′ N, 165°49.4′ E, 289 m, silt over rocky ground	8.9	3.1

**Table 2:** Biological activity of aqueous/ethanol extracts of sea anemones.

Species	Hemolytic Activity, MC_100_ ± SE	Cytotoxic Activity		Antimicrobial Activity, MC_10_ ± SE		
		Murine Splenocytes, MC_70_ ± SE, μg	Ehrlich Carcinoma Cells, MC_70_ ± SE, μg	*B*	*S*	*C*
*Stomphia coccinea*	15.3 ± 0.05/–	–/–	15.3 ± 0.09/14.5 ± 0.10 *	8.5 ± 0.03/–	8.5 ± 0.06/–	8.5 ± 0.12/–
*Actinostola callosa*	7.6 ± 0.12/–	–/–	7.6 ± 0.04 **/7.5 ± 0.06 *	–/–	–/12.5 ± 0.09	–/–
*Actinostola faeculenta*	35.5 ± 0.01/–	–/9.3 ± 0.02	35.5 ± 0.8 */9.3 ± 0.05 *	–/–	–/–	–/–
*Liponema brevicorne* (Kuril Islands)	8.8 ± 0.04/–	8.8 ± 0.09 */–	–/–	4.6 ± 0.08/–	–/–	–/–
*Liponema brevicorne* (Commander Islands)	–/7.6 ± 0.12	–/–	–/–	–/–	–/–	–/–
*Corallimorphus* cf. *pilatus* (Kuril Islands)	15.0 ± 0.04/45.0 ± 0.08	–/–	15.0 ± 0.25 **/9.0 ± 0.08	10.0 ± 0.04/–	10.0 ± 0.08/–	10.0 ± 0.03/–
*Corallimorphus* cf. *pilatus* (Commander Islands)	–/–	–/–	–/31.0 ± 0.06	–/–	–/–	–/–

* MC_30_; ** MC_50_; –, no activity; SE, standard error; *B*, *Bacillus subtilis*; *S*, *Staphylococcus aureus*; *C*, *Candida albicans*; MC_10_, lack of bacteria growth till 10 mm.

**Table 3:** Activity of aqueous and ethanol extracts of sea anemones against α-galactosidases.

Species	Residual Activity, % ± SE			
	Ethanol Extract		Aqueous Extract	
	α-galactosidase	α-galactosidase C494N	α-galactosidase	α-galactosidase C494N
*Stomphia coccinea*	42 ± 1.3	100 ± 0.03	147 ± 0.65	N.d.
*Actinostola callosa*	45 ± 0.8	50 ± 1.8	174 ± 0.9	N.d.
*Actinostola* *faeculenta*	20 ± 1.12	30 ± 1.04	148 ± 1.1	N.d.
*Liponema* *brevicorne* (Kuril Islands)	6 ± 1.0	N.d.	126 ± 0.95	N.d.
*Liponema* *brevicorne* (Commander Islands)	113 ± 0.93	N.d.	115 ± 0.78	N.d.
*Corallimorphus* cf. *pilatus* (Kuril Islands)	37 ± 1.6	50 ± 0.18	170 ± 0.92	N.d.
*Corallimorphus* cf. *pilatus* (Commander Islands)	61 ± 1.37	N.d.	117 ± 1.2	N.d.

N.d., not determined; SE, standard error.

**Table 4:** Peptide molecular masses found in *L. brevicorne* and *C.* cf. *pilatus* fractions.

Fractions	Peptide Molecular Masses Found in Aqueous Extracts, Da		Peptide Molecular Masses Found in Ethanol Extracts, Da	
	*L. brevicorne*	*C.* cf. *pilatus*	*L. brevicorne*	*C*. cf. *pilatus*
1	4050, 4221, 4504 5545 7864 8006 9884 10,395	2277 3264, 3391, 3582, 3835 4102, 4336, 4542, 4882 5153, 5539 6135, 6763, 6973 8241, 8393, 8548, 8896 9023, 9095, 9805	1527 2982 3052, 3182, 3403, 3947 4034, 4477, 4850 5053, 5274, 5381, 5522, 5934 8603 9785	2984 3075, 3586, 3780, 3865 4101, 4186, 4314, 4430, 4542, 4615, 4770, 4882 5151, 5410, 5769 8486, 8564, 8779, 8897 9121, 9808
2	2054, 2868, 2982 3198, 3663 4122, 4253, 4501 5111, 5415, 5970 7468 8322, 8469, 8600	3585, 3781, 3888 4030, 4337, 4444, 4543, 4882 5153, 5774, 5890 6136, 6370 8177, 8288, 8470, 8577, 8690, 8779, 8898 9100, 9812	2885, 2890 3829 4044, 4725, 4955, 4960 5091, 5182, 5267, 5271, 5406, 5547, 5549, 5752 9124 10,607, 10,846	3053, 3273, 3570, 3781 4021, 4086, 4187, 4306, 4457, 4560, 4600, 4743 5317, 5378, 5463, 5555, 5659, 5796, 5886
3	1458, 1687, 1950 2186, 2402, 2618, 2639, 27,132,789 3257, 3681, 3783, 3787, 3957 4078, 4132, 4242, 4725, 4780, 4806, 4956, 4970 5188, 5190, 5275, 5309, 5316, 5403, 5415, 5547, 5554, 5749, 5757, 5969 9768	1298, 1491 2123, 2978 3026, 3144, 3342, 3695, 3779 4020, 4023, 4186, 4302, 4376, 4383, 4539, 4542, 4873 5184, 5272, 5275, 5552, 5773, 5787, 5888, 5948 5959	1517, 1737, 1969 2215, 2492, 2970 3196, 3449, 3841 4039, 4727, 4944 5307, 5550, 5774	5546, 5760
4	1482, 1723, 1986 2214, 2455, 2719, 2971 3191 4784 5548, 5765, 5884	1520, 1720, 1993 2525 3974 4125, 4542, 4738 5231, 5374 6025, 6946 7608		

## Data Availability

Sequences of 18S, COI, and ITS genes are available in the GenBank database under accession numbers OK642140-OK642144, OK638712-OK638716, and OL329854-OL329860, respectively. The additional data support the manuscript are available from the corresponding author upon request.

## Supplementary Materials

The following are available online at https://www.mdpi.com/article/10.3390/md19120654/s1, Figure S1: NJ phylogenetic trees of sea anemones from suborder Nynantheae inferred from (a) COI and (b) ITS gene sequences; Table S1: List of identified proteins from ethanol fraction 3 of *L. brevicorne* using Actiniaria protein database.
