# Supplementary figures and images for: Deep-Sea Anemones Are Prospective Source of New Antimicrobial and Cytotoxic Compounds
Source: Mar Drugs. 2021 Nov 24;19(12):654. doi: 10.3390/md19120654 (PMC8704684; doi:10.3390/md19120654)

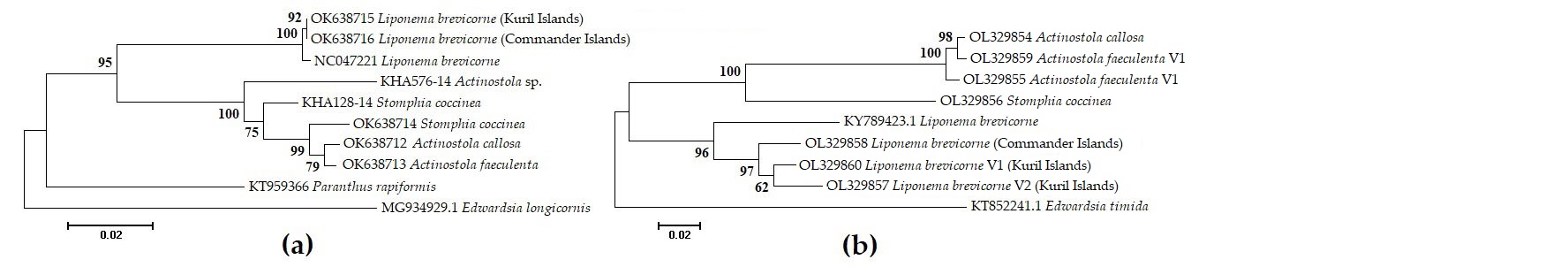

Supplement: Supplementary file 1 [file marinedrugs-19-00654-s001.zip › Kvetkina_Figure S1.jpg]
